# Comparison of phase dyssynchrony analysis using gated myocardial perfusion imaging with four software programs: Based on the Japanese Society of Nuclear Medicine working group normal database

**DOI:** 10.1007/s12350-015-0333-y

**Published:** 2016-02-09

**Authors:** Kenichi Nakajima, Koichi Okuda, Shinro Matsuo, Keisuke Kiso, Seigo Kinuya, Ernest V. Garcia

**Affiliations:** 1 Department of Nuclear Medicine, Kanazawa University Hospital, 13-1 Takara-machi, Kanazawa, 920-8641 Japan; 2 Department of Physics, Kanazawa Medical University, Uchinada, Japan; 3 Department of Radiology, National Cardiovascular Center, Suita, Japan; 4 Department of Radiology and Imaging Sciences, Emory University, Atlanta, GA USA

**Keywords:** Gated myocardial perfusion image, normal database, phase, dyssynchrony, Japanese Society of Nuclear Medicine working group

## Abstract

**Purpose:**

Left ventricular (LV) phase dyssynchrony parameters based on gated myocardial perfusion imaging varied among software programs. The aim of this study was to determine normal ranges and factors affecting phase parameters.

**Methods:**

Normal databases were derived from the Japanese Society of Nuclear Medicine working group (*n* = 69). The programs were Emory Cardiac Toolbox with SyncTool (ECTb), Quantitative Gated SPECT (QGS), Heart Function View (HFV), and cardioREPO (cREPO); parameters of phase standard deviation (PSD), 95% bandwidth, and entropy were compared with parameters with ECTb as a reference.

**Results:**

PSD (degree) was 5.3 ± 3.3 for QGS (*P* < .0001), 5.4 ± 2.5 for HFV (*P* < .0001), and 10.3 ± 3.2 for cREPO (*P* = n. s.) compared with 11.5 ± 5.5 for ECTb. Phase bandwidth with three programs differed significantly from ECTb. Gender differences were significant for all programs, indicating larger variation in males. After adjustment of LV volumes between genders, the difference disappeared except for QGS. The phase parameters showed wider variations in patients with the lower ejection fraction (EF) and larger LV volumes, depending on software types.

**Conclusion:**

Based on normal ranges of phase dyssynchrony parameters in four software programs, dependency on genders, LV volume, and EF should be considered, indicating the need for careful comparison among different software programs.

## Introduction

In nuclear cardiology, phase analysis of timing of contraction has been used since the 1980s in a gated blood-pool study.^1^ Fourier analysis of pixel-based time-activity curves provided phase and amplitude of the fundamental frequency, and it has been used for quantifying regional ventricular wall motion.^2^,^3^ Using a combination of phase and amplitude, hypokinesis was defined as decreased amplitude with a normal phase, akinesis as severely reduced (nearly zero) amplitude, and dyskinesis as delayed phase. In addition, phase propagation sequence analysis in conditions such as bundle branch block, ventricular pacing, and pre-excitation syndromes can be characterized by the specific propagation patterns of phases in both ventricles in planar and tomographic studies.^4^-^7^ However, after the advent of gated myocardial perfusion imaging (MPI) along with quantitative gated single-photon emission computed tomography (SPECT) software as Quantitative Gated SPECT (QGS; Cedars Sinai Medical Center, USA) and Emory Cardiac Toolbox (ECTb, Emory University, USA),^8^,^9^ the use of gated blood-pool studies was decreased and the phase analysis lost its popularity. ECTb-SyncTool (Syntermed, USA) successfully used phase analysis in gated SPECT studies as commercially available software.^10^

Since several software packages are now available in Japan, the purpose of this study was to determine the normal values of each software program, including cardioREPO (cREPO; FUJIFILM RI Pharma, Tokyo, Japan; collaboration with EXINI Diagnostics, Lund, Sweden) and Heart Function View (HFV; Nihon Medi-Physics, Tokyo, Japan) in comparison with ECTb. The normal database was prepared from the Japanese Society of Nuclear Medicine (JSNM) working group database 2007.^11^,^12^

## Methods

### Databases Used for the Analysis

The JSNM working group created normal databases for MPI in 2007, and the electrocardiographic gated ^99m^Tc-methoxy-isobutylisonitrile (MIBI) or ^99m^Tc-tetrofosmin studies at resting condition with 16 frames per cardiac cycle were used.^11^ A standard dose of 555 to 925 MBq in Japan was used in all institutions. A total of 69 datasets were available from the database (36 male and 33 female subjects), which were obtained by rotating Anger camera system. All the patients showed no perfusion defect as assessed by visual and quantitative scoring with summed score of ≤3 with a 17-segment 5-point (0-4) model. The databases included subjects without underlying cardiac diseases and no medications for diabetes and hypertension. Subjects with inappropriate arrhythmia for gating and wall motion abnormality were excluded. Mean age was 56 ± 13 years including 36 male and 33 female subjects. Body mass index was 22.5 ± 2.6 and 22.7 ± 2.7 for male and female subjects, respectively. All the images were 64 × 64 matrices and acquired with either 180° or 360° rotation. The databases were prepared as a short-axis image set of gated and non-gated data. The gated short-axis images at rest showed sufficient maximum myocardial count of 123 ± 64 counts/pixel. No study showed visual flickering of the data due to count drop of the last frames. The reconstruction parameters for MPI were standardized as described elsewhere.^11^ All the MPI data were reconstructed by the filtered back-projection method. Neither attenuation correction nor scatter correction was used.

### Left Ventricular (LV) Ejection Fraction (EF) and Volumes for Gated SPECT Study

Parameters for baseline cardiac function included left LVEF, end-diastolic volume (EDV), and end-systolic volume (ESV). All the analyses were performed using standard software settings of ECTb, QGS, HFV, and cREPO. Regarding ventricular edge detection, ECTb used an anatomically based 3-dimensional model for ventricular edge detection assuming that at end-diastole, LV myocardial thickness is 1 cm.^9^,^13^ QGS software used an ellipsoid shape and iterative process to fit the myocardial walls.^8^,^14^ The algorithm of HFV used the “modified threshold method”,^15^ and the method of cREPO based on the active-shape model.^16^,^17^ All of these processing algorithms were specifically developed and refined thereafter.^13^,^14^ Software versions used in this study were version 2008 for QGS, version 3.2 for ECTb, version 1.0 for cREPO, and version 1.1 for HFV.

### Dyssynchrony Analysis

**Figure 1:**
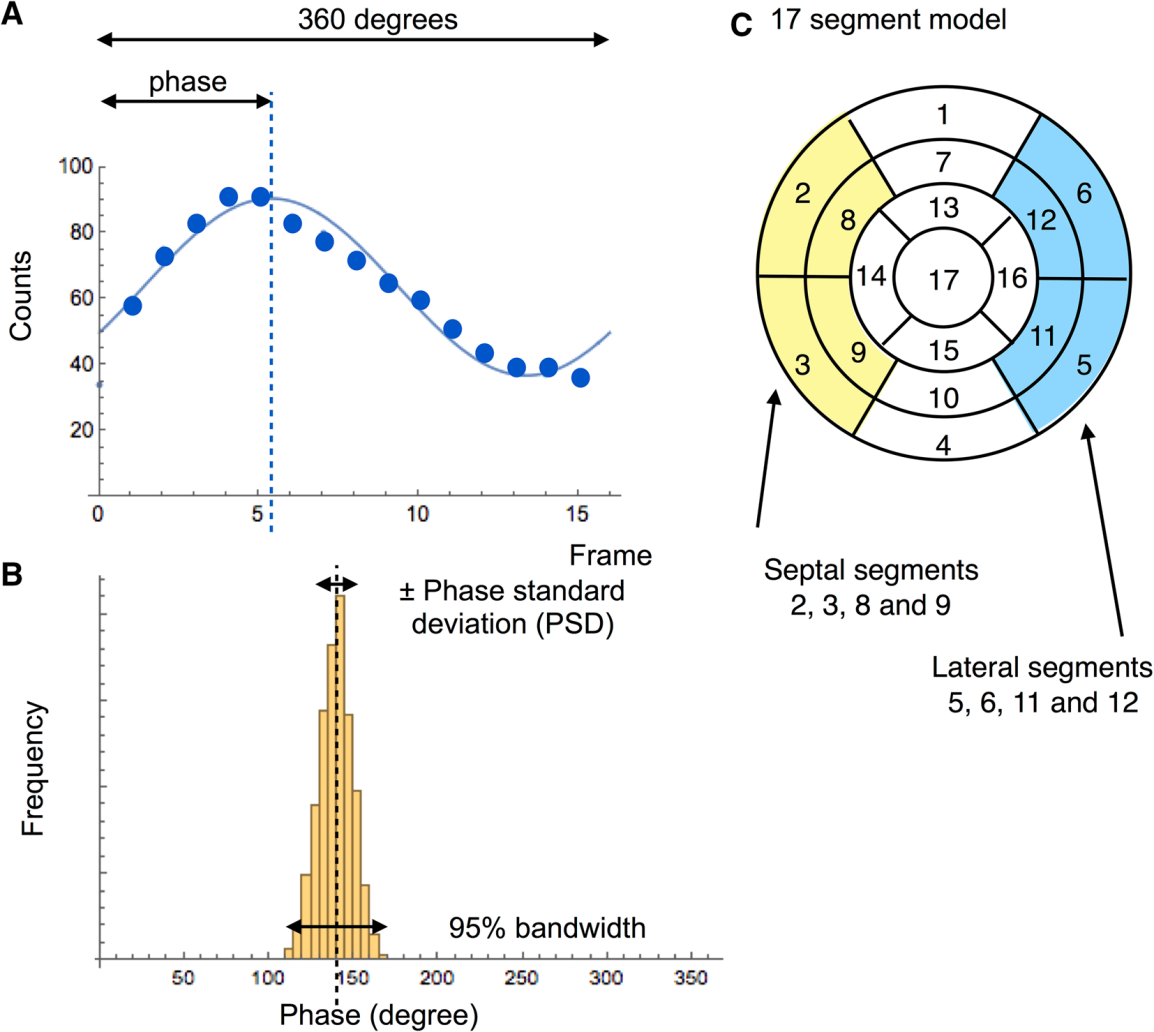
Parameters calculated with gated SPECT study. A phase value is calculated based on original data points (**A**). A histogram is created to obtain PSD and 95% bandwidth (**B**). Septal and lateral segments are shown to calculate variations of time to end-systole (**C**)

Fourier analysis was applied to extract the phase and amplitude of the fundamental frequency of the regional time-activity curves.^1^,^18^ In gated MPI, the activity change reflects wall thickening during a cardiac cycle due to partial volume effects. Although the precise algorithm for calculating regional curves and segmentation differed among software programs, the following parameters have been commonly used. The phase value was defined as an amount of shift of sine (or cosine) curves using the fundamental frequency of the Fourier fitting (Fig. 1A), and a phase histogram was created to see the distribution pattern of phase values. Based on the histogram analysis, standard deviation of the phase (PSD, unit: degree) and 95% width of the histogram or bandwidth (unit: degree) were calculated (Fig. 1B). Entropy is an index of “disorder” defined by summation of [*f*_i_*log(*f*_*i*_)]/Log(*n*)], where *f* and *n* are frequency in the *i*_th_ bin and number of bins, respectively.^19^,^20^ The entropy shows a range of 0-1 (0-100%), corresponding to complete order to disorder. All the analyses were performed automatically at first, and when statistical noise was included in the basal part of the phase polar map, manual adjustment of the base was added. Phase map smoothing function was used for ECTb, and basal circular removal function was used for cREPO when they were required. Minor manual adjustment of the basal border was performed in <10% of the patients for QGS, cREPO, and HFV, and ~30% for ECTb.

In addition, cREPO provided 17-segment-based regional time-activity curves and its regional variations similar to the “cardioGRAF” software (FUJIFILM RI Pharma, Co. Ltd, Japan), which has been used for heart failure and resynchronization therapy.^21^,^22^ After calculating regional counts in each segment, 17 time-activity curves were created to calculate time to the end-systolic frame (TES, unit: degree) using the fundamental wave plus the second harmonic component. The maximum difference among segmental TES (MDTES: unit, %) was calculated after division by 360°. The standard deviation of TES (SDTES) was also calculated (unit, %). The difference of TES between the lateral segment (average of anterolateral and inferolateral segments) and the septum (average of anteroseptal and inferoseptal segments) (DTES-LS: unit, %) was calculated (Fig. 1C).

### Statistical Analysis

All the data are expressed as mean and standard deviation (SD). Mean values were compared by the analysis of variance. Scatterplot matrix was calculated for all combinations of the four software programs, and pairwise correlation coefficients and p values were calculated. Gender difference was also calculated for all phase parameters. Linear regression line was calculated with the least square method. To compare parameters for gender differences in the small and larger LV, non-parametric Wilcoxon test was used for the four groups, and only males with the smaller LV and females with the larger LV were compared. Statistical analysis was performed using JMP 10.0 software (SAS Institute Inc., Cary, NC, USA). A *P* value <.05 was considered significant.

## Results

The mean EF was 69.2 ± 6.5%, 77.1 ± 6.3%, 73.2 ± 5.3%, and 71.7 ± 6.4% for QGS, ECTb, cREPO, and HFV, respectively (Table 1). Both EDV and ESV were the highest in cREPO. For all pairwise comparisons of EF, the correlation coefficient ranged from 0.53 (QGS vs cREPO) to 0.76 (HFV vs cREPO) (*P* < .0001 for all combinations).

**Table 1:** EF and volumes in JSNM working group normal database

	All mean ± SD (*n* = 69)	*P* value vs QGS	Male mean ± SD (*n* = 36)	Female mean ± SD (*n* = 33)	*P* value male vs female
EF (%)					
QGS	69.2 ± 6.5		66.5 ± 6.1	72.0 ± 5.7	.0002
ECTb	77.1 ± 6.3	<.0001	74.3 ± 5.4	80.3 ± 5.7	<.0001
HFV	71.7 ± 6.4	.024	69.5 ± 6.4	74.0 ± 5.7	.003
cREPO	73.2 ± 5.3	.0001	72.1 ± 5.4	74.5 ± 5.1	.061
EDV (mL)					
QGS	74.7 ± 17.3		84.4 ± 15.7	64.1 ± 12.1	<.0001
ECTb	87.7 ± 20.2	<.0001	98.1 ± 19.3	76.3 ± 14.3	<.0001
HFV	77.7 ± 17.7	.32	87.6 ± 15.8	66.8 ± 12.5	<.0001
cREPO	90.4 ± 19.5	<.0001	100.9 ± 18.0	78.9 ± 13.8	<.0001
ESV (mL)					
QGS	23.8 ± 9.4		28.8 ± 9.2	18.2 ± 6.0	<.0001
ECTb	20.7 ± 9.1	.051	25.6 ± 8.8	15.3 ± 5.8	<.0001
HFV	22.5 ± 8.8	.40	27.1 ± 8.8	17.5 ± 5.4	<.0001
cREPO	24.1 ± 6.7	.83	27.9 ± 6.0	19.9 ± 4.5	<.0001

*JSNM*, Japanese Society of Nuclear Medicine; *PSD*, phase standard deviation; *ECTb*, Emory Cardiac Toolbox with SyncTool; *QGS*, Quantitative Gated SPECT; *HFV*, Heart Function View; *cREPO*, cardioREPO

Regarding phase parameters (Table 2), the bandwidth was the highest in cREPO, followed by ECTb, QGS, and HFV (*P* < .0001 vs ECTb for all). The PSD was comparable for ECTb and cREPO (*P* = n. s.), which was higher than that of QGS (*P* < .0001) and HFV (*P* < .0001). Phase entropy was higher in cREPO than that in QGS (*P* < .0001).

**Table 2:** Phase parameters in JSNM working group normal database

	Mean ± SD	Lower–upper limits	*P* value vs ECTb
Phase bandwidth (°)			
ECTb	29.4 ± 9.3	11–49	
QGS	21.9 ± 8.6	5–39	<.0001
HFV	19.9 ± 9.1	2–38	<.0001
cREPO	40.3 ± 11.6	17–64	<.0001
PSD (°)			
ECTb	11.5 ± 5.5	1–23	
QGS	5.3 ± 3.0	0–11	<.0001
HFV	5.4 ± 2.5	0–10	<.0001
cREPO	10.3 ± 3.2	4–17	.12
Phase entropy (%)			
QGS	24.0 ± 8.3	7–41	
cREPO	43.0 ± 6.4	30–56	<.0001 vs QGS

Upper and lower limits are given by mean ± 2SD

*PSD*, phase standard deviation; other abbreviations are the same as in Table 1

**Figure 2:**
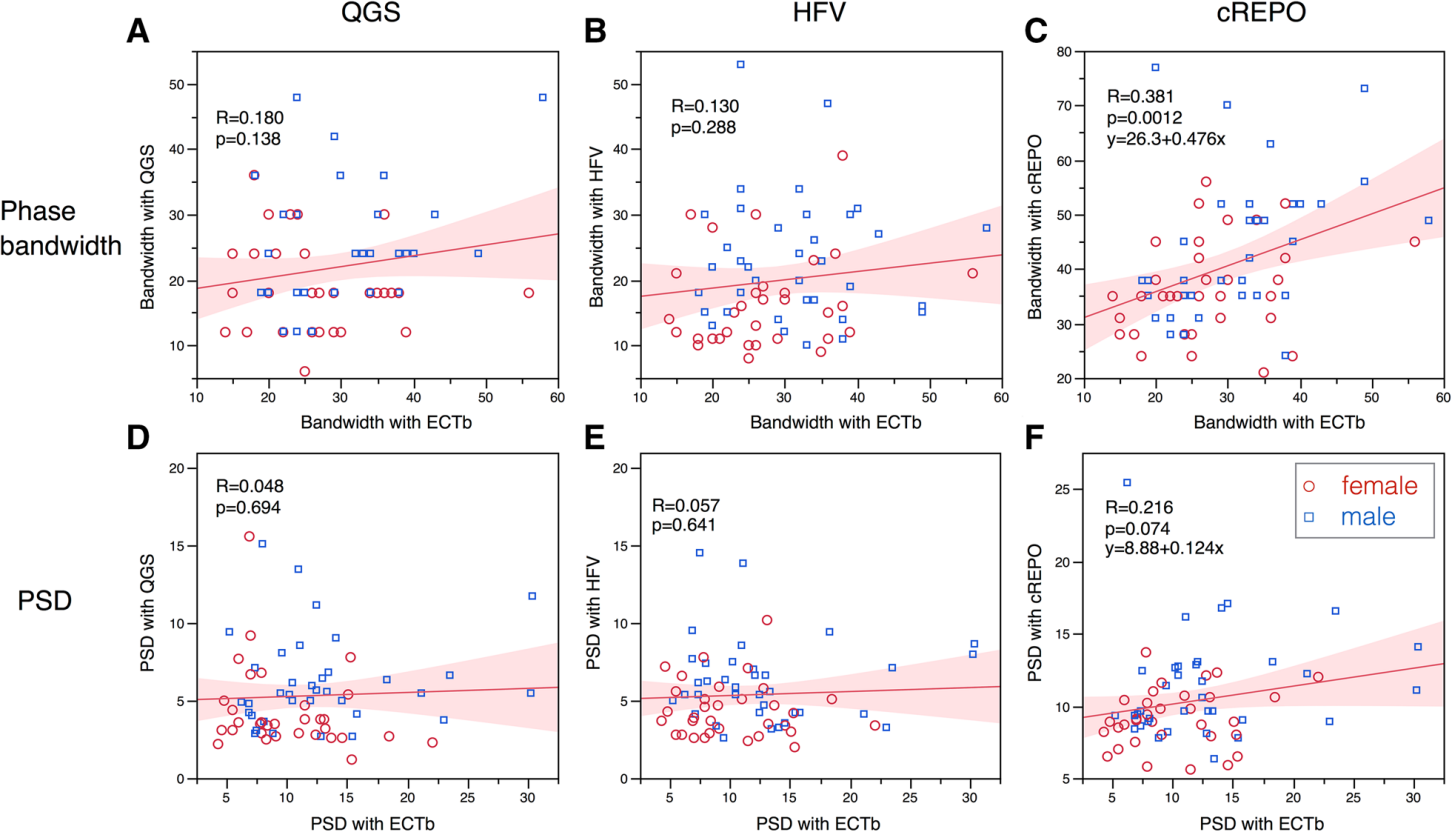
Relationship of phase bandwidth and PSD compared with those with ECTb. * Upper panels* show bandwidth in QGS, HFV, and cREPO vs ECTb.* Lower panels* show PSD between two software programs. The linear regression equation is written when the *P* value is <.10. The * shaded area* indicates confidence limits for the regression line. * Red circles* and * blue squares* denote male and female data points, respectively

**Figure 3:**
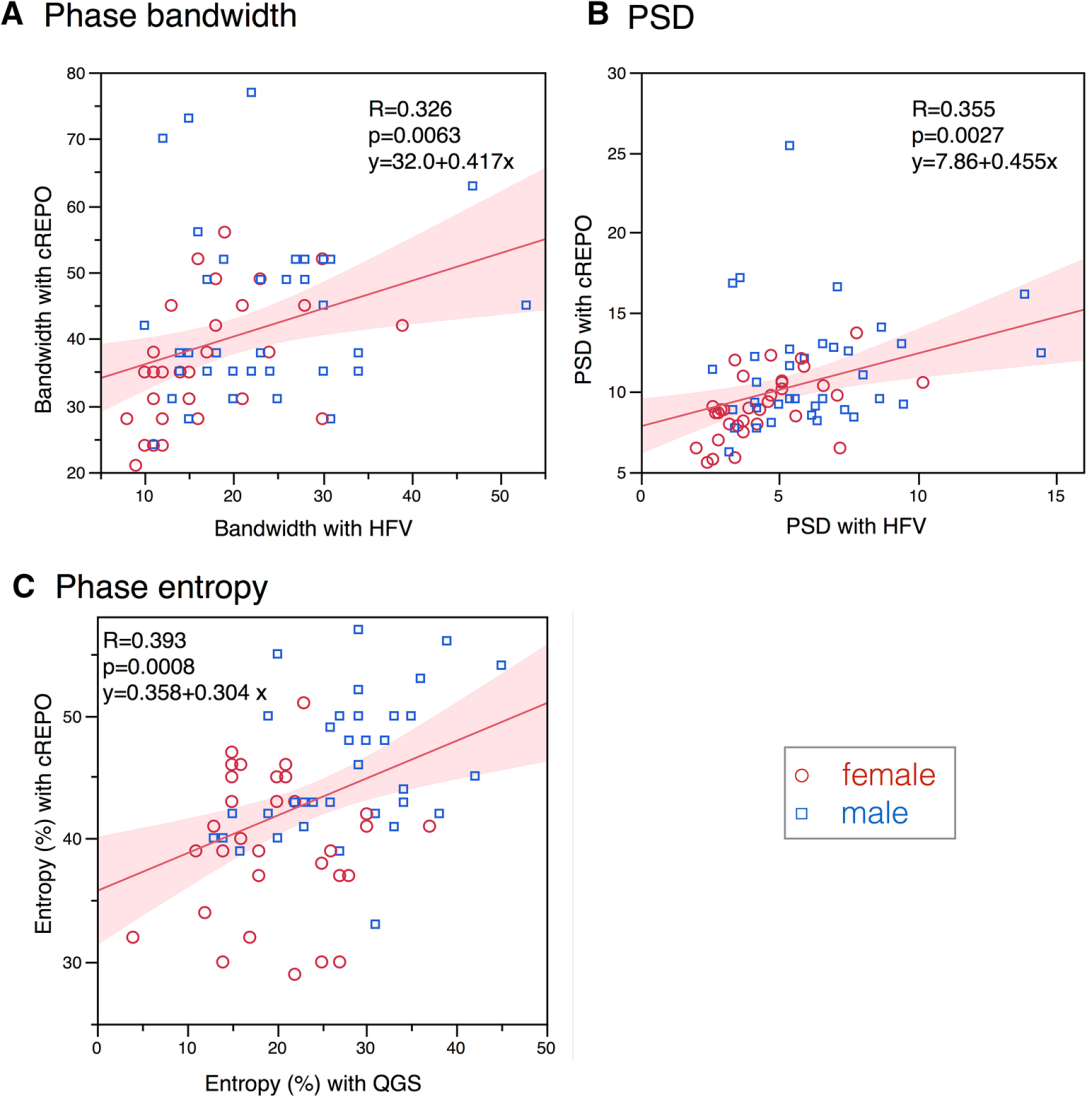
Relationship of phase bandwidth, PSD, and phase entropy between two software programs. * Panels* (**A**) and (**B**) show bandwidth and PSD for HFV vs cREPO, and * panel* (** C**) shows phase entropy for QGS vs cREPO. Marks and lines are the same as in Fig. 2

When pairwise comparison was performed for the phase bandwidth, significant correlation was observed between ECTb and cREPO [*r* = 0.38, 95% confidence interval (CI) = 0.16-0.57, *P* = .0012] (Fig. 2C), and between cREPO and HFV (*r* = 0.33, CI 0.10-0.52, *P* = .0063) (Fig. 3A). Similarly, for PSD, significant correlation was observed between cREPO and HFV (*r* = 0.36, CI 0.13-0.55, *P* = .0027) (Fig. 3B). The phase entropy showed significant correlation between QGS and cREPO (*r* = 0.39, CI 0.17-0.58, *P* = .0008) (Fig. 3C). Other combinations did not show significant correlation, since all values were in normal ranges.

**Figure 4:**
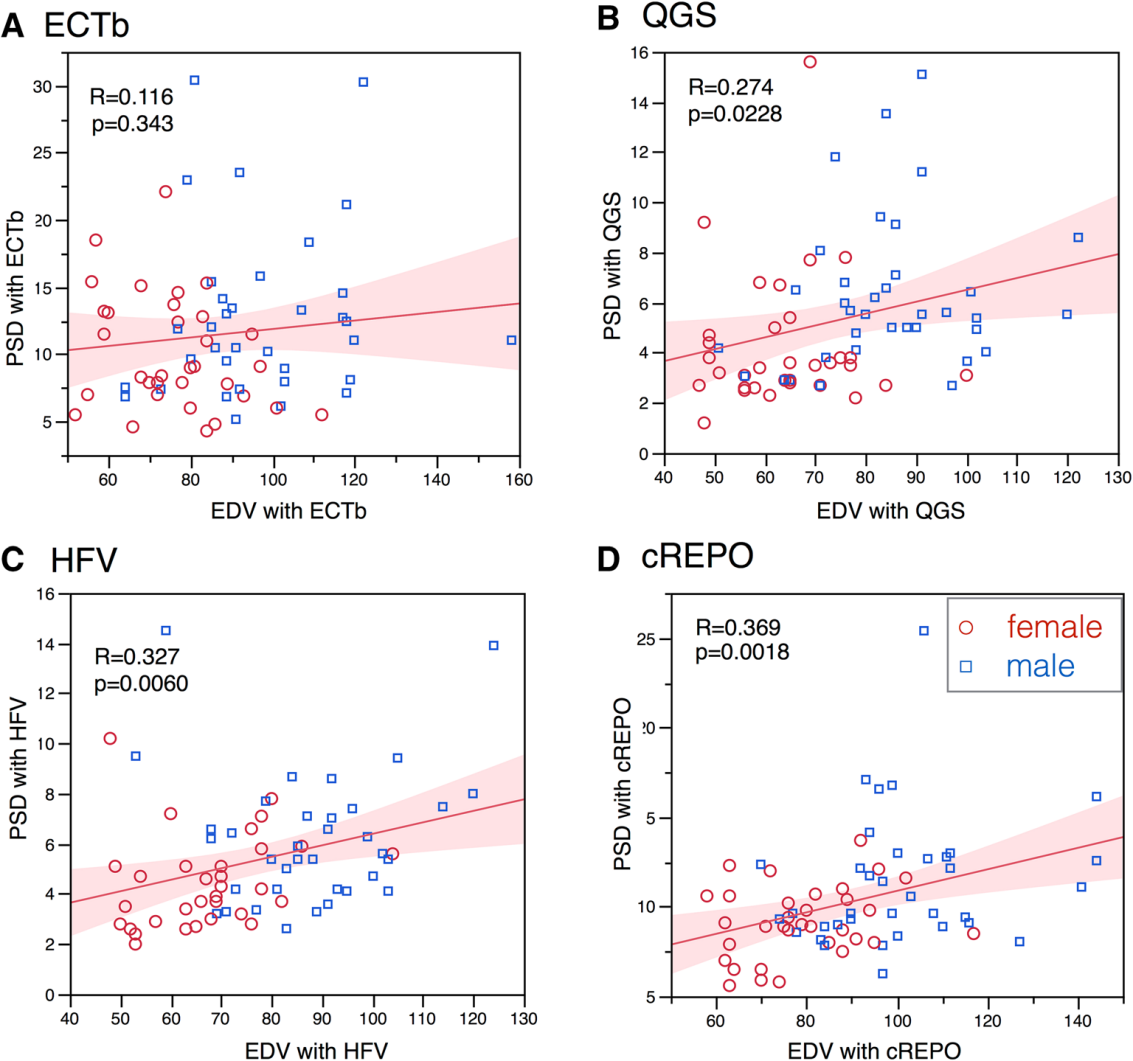
Relationship between EDV and PSD using ECTb (**A**), QGS (**B**), HFV (**C**), and cREPO (**D**). The * shaded area* indicates confidence limits for the regression line. * Marks* and * lines* are the same as in Fig. 2

**Figure 5:**
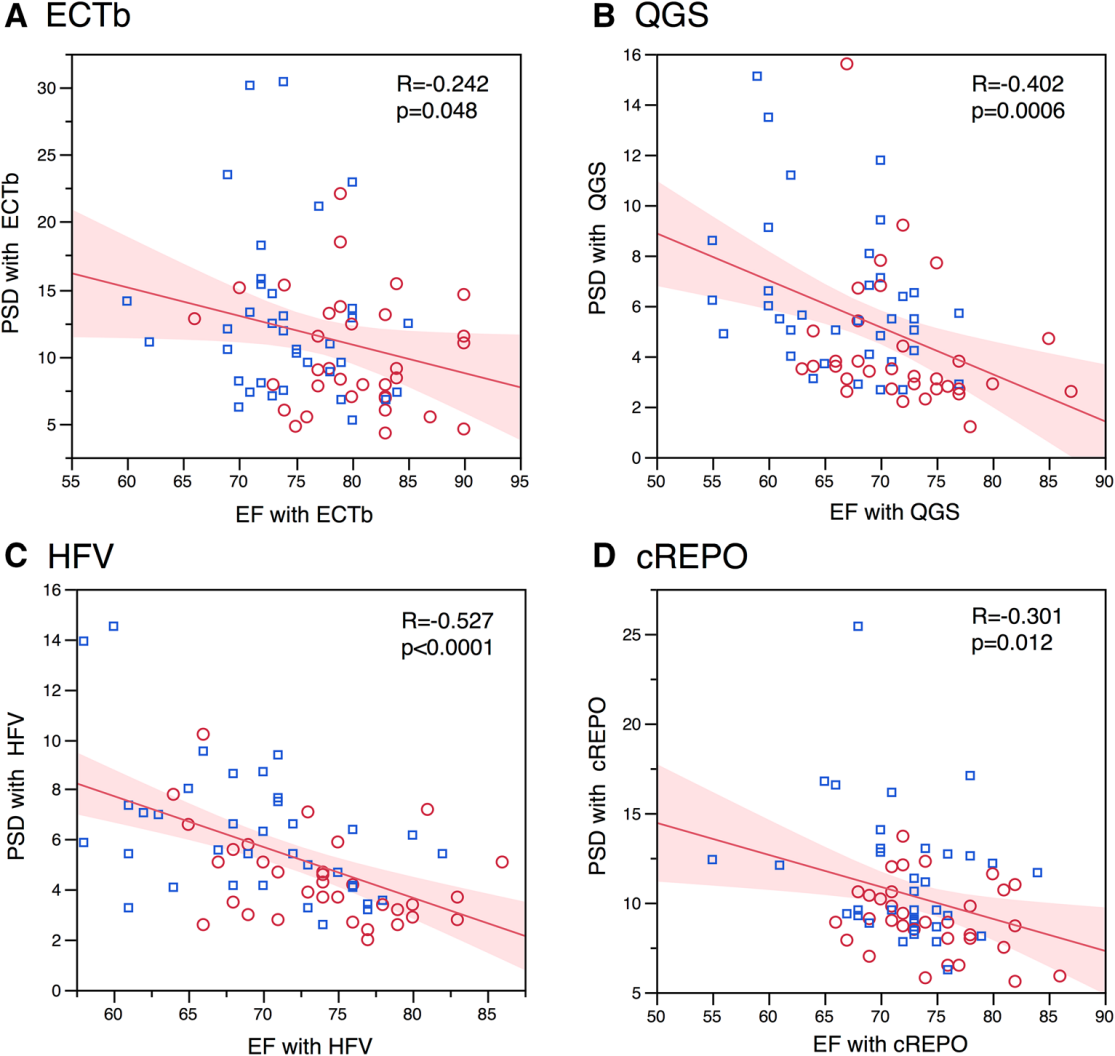
Relationship between LVEF and PSD using ECTb (**A**), QGS (**B**), HFV (**C**), and cREPO (**D**). The * marks* and * lines* are the same as in Fig. 2

Gender difference of phase parameters was significant, and male subjects showed larger phase deviations (Table 3). In the phase bandwidth, QGS, cREPO, and HFV showed higher values in males than in females (*P* = .0014-.010), and ECTb also showed higher tendency in males (*P* = .078). The PSD was consistently higher (*P* = .002-.043) in males than in females. The phase entropy parameter was also higher in males, indicating larger disorder in the male database (*P* < .0001). When phase bandwidth and PSD were plotted versus EDV, positive correlations were obtained for QGS, HFV, and cREPO (Fig. 4). The relationship between phase parameters and LVEF was examined. Regarding phase bandwidth, significant correlation with LVEF was observed in QGS (*r* = −0.46, *P* < .0001) and HFV (*r* = −0.55, *P* < .0001). The relationship between PSD and LVEF showed that correlation coefficients ranged from −0.24 to −0.53 (*P* < .0001 to .048) (Fig. 5). Phase entropy and LVEF showed negative correlation: −0.44 (*P* = .0002) in QGS and −0.31 (*P* = .009) in cREPO. However, the relationship between phase parameters and body weight were not significant in all software programs. Parameters of TES using a 17-segment model were given only with cREPO (Table 4). Although the values were slightly higher in males, no significant gender difference was observed.

**Table 3:** Phase parameters in JSNM working group normal database in male and female subjects

	Male mean ± SD (*n* = 36)	Lower–upper limit	Female mean ± SD (*n* = 33)	Lower–upper limit	*P* value male vs female
Phase bandwidth (°)					
ECTb	31.3 ± 9.4	13–50	27.3 ± 8.9	10–45	.078
QGS	25.0 ± 8.9	7–43	18.5 ± 6.9	5–32	.0014
HFV	23.1 ± 9.5	4–42	16.5 ± 7.2	2–31	.0018
cREPO	43.7 ± 12.8	18–69	36.6 ± 9.0	19–54	.010
PSD (°)					
ECTb	12.8 ± 6.2	0–25	10.1 ± 4.3	2–19	.043
QGS	6.2 ± 3.0	0–12	4.3 ± 2.7	0–10	.0065
HFV	6.2 ± 2.7	1–12	4.4 ± 1.8	1–8	.0020
cREPO	11.4 ± 3.7	4–19	9.1 ± 2.0	5–13	.0029
Phase entropy (%)					
QGS	27.8 ± 7.8	12–43	19.8 ± 6.7	6–33	<.0001
cREPO	45.9 ± 5.6	35–57	40.0 ± 5.8	28–52	<.0001

Abbreviations are the same as in Table 1

**Table 4:** Parameters using time to end-systole (TES) and 17-segment regional analysis

	Total mean ± SD (*n* = 69)	Male mean ± SD (*n* = 36)	Female mean ± SD (*n* = 33)	*P* value male vs female
MDTES (%)	8.75 ± 3.39	9.29 ± 3.60	8.17 ± 3.10	.17
SDTES (%)	2.26 ± 0.81	2.37 ± 0.89	2.15 ± 0.70	.25
DTES-LS (%)	1.80 ± 1.35	1.88 ± 1.58	1.71 ± 1.06	.61

*MDTES*, maximum difference in TES; *SDTES*, standard deviation of TES; *DTES-LS*, difference in TES between the lateral wall and septum

To evaluate the effect of small hearts on phase parameters, male subjects with the lower LV volume (EDV <85 mL, median value) and female subjects with the higher LV volumes (EDV ≥65 mL, median value) were compared (Table 5). Although both EDV and EF calculated by QGS software did not differ significantly, PSD (*P* = .040) and entropy (*P* = .020) showed significant difference between genders with QGS. A slightly higher tendency (*P* = .05 to .010) was observed in males than females regarding PSD by ECTb, bandwidth by QGS, and bandwidth and PSD by HFV.

**Table 5:** Comparison of phase parameters between female subjects with large LV and male subjects with small LV

	Males with smaller LVEDV <85 mL	Females with larger LVEDV ≥65 mL	*P* value*
N	19	16	
EDV (mL)	73.1 ± 9.2	73.7 ± 9.0	.58
ESV (mL)	23.5 ± 6.3	22.4 ± 4.7	.84
EF (%)	68.2 ± 5.9	69.6 ± 4.2	.76
ECTb			
Phase bandwidth (°)	31.0 ± 10.0	25.9 ± 6.8	.17
PSD (°)	12.8 ± 6.6	9.2 ± 3.4	.091
QGS			
Phase bandwidth (°)	23.7 ± 8.6	19.1 ± 6.3	.084
PSD (°)	6.0 ± 3.0	4.7 ± 3.4	.040
Entropy (%)	26.3 ± 8.0	20.1 ± 5.5	.020
HFV			
Phase bandwidth (°)	23.4 ± 10.2	17.9 ± 6.0	.097
PSD (°)	6.3 ± 2.8	4.8 ± 1.6	.098
cREPO			
Phase bandwidth (°)	39.0 ± 8.3	38.9 ± 8.0	.97
PSD (°)	10.2 ± 2.6	9.6 ± 1.7	.62
Entropy (%)	43.8 ± 5.3	42.1 ± 4.0	.29

* Non-parametric Wilcoxon test; 85 and 65 mL are median values of each gender

## Discussion

When Fourier phase analysis has been used in gated myocardial SPECT studies, it has been noticed that normal values depended on the technology of data acquisition and processing as well as software algorithms. Since new software, namely, cREPO and HFV has been available in Japan, normal values based on JSNM working group databases are presented and compared with more commonly used software of ECTb and QGS. In this comparative study of normal values, phase parameters differ significantly among software programs and are not considered to be interchangeable. Both phase bandwidth and PSD were higher in ECTb and cREPO than in QGS and HFV. Gender differences and dependency on the LV volume and LVEF should also be considered for clinical applications.

Phase analysis was developed in the 1980s and has been used in gated blood-pool study and subsequently in gated SPECT. Initial applications were to detect ventricular asynergy and conduction abnormalities including bundle branch block and pre-excitation syndrome.^1^-^3^,^5^,^7^ However, Fourier phase analysis has been used for gated MPI, which used regional myocardial count. The difference in these methods is that the blood-pool phase analysis yields insight into wall motion, while MPI phase analysis focuses on the timing and amplitude of wall thickening or count changes. Chen and Garcia developed the first successful software, and the results have been found to be promising in LV mechanical dyssynchrony analysis and cardiac resynchronization therapy (CRT).^10^,^18^ Similar software for dyssynchrony analysis has also been developed in Japan using segmental regional variation of TES, which showed that the combined use of regional variation of contraction timing and perfusion in the inferolateral segments was useful for evaluating the effect of CRT.^15^,^21^ The phase analysis was also applicable for evaluating multi-vessel disease and ischemic and non-ischemic etiologies with stress MPI.^23^,^24^

Several dyssynchrony parameters have been proposed. Although the distribution of phase values is nearly symmetrical in normal subjects, it is not a simple Gaussian distribution in patients with dyssynchrony. PSD may not be appropriate for characterizing the widely distributed and sometimes multi-modal distributions in phase histograms. The 95% bandwidth includes nearly a whole range of histogram distribution by excluding possible outlier phase values, and it has worked well in a number of studies. Therefore, we compared these common parameters of PSD and 95% bandwidth in the four software programs. The entropy, which is defined as a term of physics, was considered to be a promising parameter in gated blood-pool study for indication of CRT.^19^ Random distribution in the dilated left ventricle with reduced contractility, which cannot be reflected by simple PSD and bandwidth, may fit the use of entropy.

The fair correlation among software programs was apparently due to narrow range of distribution, since only functionally normal patients were included. If abnormal patients with LV dyssynchrony were included, correlation coefficient would be calculated as higher. Therefore, we cannot directly apply the linear regression equations that were presented in this study for converting the phase values in the patient population including the abnormal phase. The reliability of phase and cross-calibration between software types should be evaluated by including a large number of abnormal patients. Moreover, the normal upper limit should not be used to indicate the optimal threshold for resynchronization, because the cutoff point for predicting good response to CRT was higher, for example 135 degrees for histogram bandwidth and 43 degrees for PSD by ECTb.^25^

Several factors may influence the distribution of phase depending on gender, total accumulated count or noise, amount of injected radionuclide, stress or rest, and number of frames per cardiac cycle. The perfusion pattern in addition to post-stress and resting conditions were also determinants of phase values.^26^ Interestingly, all phase parameters showed the higher variation in male subjects, which has been also presented by an American population, showing 38.7 ± 11.8° and 30.6 ± 9.6° for bandwidth in males and females, respectively, and 14.2 ± 5.1° and 11.8 ± 5.2° for PSD.^18^ The reason may be related to myocardial count accumulated in various sizes of the heart. If the total injection dose was similar, the larger heart might have accumulated less myocardial count per myocardium volume (count/cm^3^). When the same acquisition and processing filters were applied in both genders, the overall effects may become smoother images in the female condition. Phase parameters showed larger variation in patients with either lower LVEF or larger LV volumes in four software programs. The difference in phase parameters became smaller between genders after adjustment of volumes, but a slight difference still remained. Generally, the higher exercise level in male subjects than in female subjects may also be a concern. Physiologically, it is not a plausible explanation that male hearts contract more dyssynchronously and disorderly than female hearts, but the gender difference actually existed in the routine acquisition and processing conditions.

Based on the results of this study, phase parameters cannot be interchangeably used among four software programs. However, when we analyzed normal values, there seem to be similarities between ECTb and cREPO, and between QGS and HFV. The ventricular models used for detecting myocardial walls significantly influence EF and volumes as well as measured pixel-based counts. The calculated phase parameters might have been influenced by the count detection algorithm on the myocardial walls, filtering of the images, number of angular sampling, filtering of time-activity curves, and so on. In general, images with smoothing or low-pass filtering, either temporal or spatial domain, result in the lower noise and hence the smaller bandwidth and PSD. Although we could not strictly define the effect confounding factors on phase values in each software program, we should carefully deal with the differences in software in clinical applications.

There are some limitations in this study. Since the JSNM working group database was prepared as the short-axis images from multiple institutions, we could not strictly define acquisition and processing conditions. However, the database reflects clinically acceptable image quality with optimal gating, and the selection criteria of the normal database were clearly defined.^11^,^12^ Since Anger camera images without attenuation correction and filtered back-projection reconstruction were used, currently used computed tomography-based attenuation correction images and new equipment such as D-SPECT could not be included. In this study, we cannot evaluate all the related factors, as the number of patients was limited in the database. Since the JSNM working group is collecting a larger number of normal databases at present, more precise analysis is indicated using the revised databases and by including diseased patients who showed abnormality in phase distribution.

## New Knowledge Gained

All normal values for phase dyssynchrony with currently available software programs were given in this study. Phase analysis parameters derived from MPI depend on software programs, due to their specific algorithms for calculating phase bandwidth, PSD, and entropy. Gender differences were observed in all software programs, and the values showed higher variations in males than in females and, in addition, in patients with the larger LV volumes and the lower LVEF. Although the results of phase analysis cannot be interchangeably used in the same subjects, such dependency on gender and functional conditions should be considered.

## Abbreviations

ECTb: Emory Cardiac Toolbox
QGS: Quantitative Gated SPECT
HFV: Heart Function View
cREPO: cardioREPO
LV: Left ventricle
PSD: Phase standard deviation
EF: Ejection fraction
EDV: End-diastolic volume
ESV: End-systolic volume
TES: Time to end-systole
